# Pilot study on the effectiveness of the socialmind program for the rehabilitation of social cognition following acquired brain injury

**DOI:** 10.3389/fpsyg.2024.1338335

**Published:** 2024-07-17

**Authors:** Sandra Rivas-García, Olga García-Bermúdez, Andrés Catena, Alfonso Caracuel

**Affiliations:** ^1^Area of Developmental and Educational Psychology, Department of Psychology, University of Cádiz, Cádiz, Spain; ^2^Mind, Brain, and Behavior Research Center-CIMCYC, University of Granada, Granada, Spain; ^3^Centro Neurons and People de Neuropsicología Infanto-juvenil, Jaén, Spain; ^4^Department of Experimental Psychology, University of Granada, Granada, Spain; ^5^Department of Developmental and Educational Psychology Department, University of Granada, Granada, Spain

**Keywords:** rehabilitation, intervention, social cognition, emotional processing, social knowledge, Theory of mind, empathy, acquired brain injury

## Abstract

**Background:**

People with acquired brain injury (ABI) often have Social Cognition (SC) deficits. Impairment of SC causes the individual to have difficulties in daily functioning and can lead to social isolation. Research aimed at rehabilitation of SC in individuals with ABI is scarce and almost always addresses only one component of this ability.

**Objective:**

This pilot study aimed to assess the effectiveness of the new “SocialMind” program in improving all core components of SC in people with ABI.

**Method:**

The study included 31 participants with ABI, divided into experimental and control groups. The study spanned 44 weeks, involving an initial meeting, evaluation, training, and final assessment phases. The SocialMind program, structured into four modules, each with a duration of 30 h, targeted each SC component through tailored exercises. The program addressed emotion recognition, social awareness, ToM, and empathy.

**Results:**

The SocialMind group demonstrated significant improvements in emotion recognition (*p* = 0.017), social knowledge (*p* < 0.001), and empathy (*p* = 0.001) compared to the control group. ToM also showed a notable improvement that approached significance (*p* = 0.057).

**Conclusion:**

This pilot study suggests that the SocialMind program effectively enhances three of the four core components of SC in individuals with ABI.

## 1 Introduction

Humans are inherently social creatures, and effective interaction with others and the environment is essential for holistic development (Fiske and Taylor, 2008; Atenas et al., 2019). Social cognition (SC) encompasses the various skills that enable such social development. SC allows individuals to perceive, understand, and interpret both their own and others’ intentions, feelings, and thoughts while facilitating the generation of adaptive social behaviors to navigate social interactions (Adolphs, 2010; McDonald and Genova, 2021) flexibly.

SC is a multifaceted skill composed of at least four core components: emotional processing, social knowledge, theory of mind (ToM), and empathy (Cassel et al., 2016). As delineated by Cassel et al. (2016) model, these components are acquired hierarchically, with cultural factors exerting an influence on their acquisition. Hence, antecedent mastery of the preceding components is imperative for the cultivation of empathy. Emotional processing is the capacity to perceive and utilize emotions effectively (Hall et al., 2018). Social knowledge involves the ability to decode and interpret behavior in specific social situations, including understanding social rules, roles, goals, and how these factors influence the behavior of others (Lima et al., 2020). ToM is the ability to comprehend the mental states of others, including their interests, beliefs, emotions, and intentions, to predict their actions, and recognize that these mental states may deviate from one’s own and reality (Byom and Mutlu, 2013). Empathy refers to the capacity to understand, share, and respond to others’ emotional experiences by adapting one’s own behavior to provide a flexible response (Laghi et al., 2019).

Key approaches can be identified to help understand the connectivity and development of these elements. Ochsner (2008) proposes that the assimilation of SC components occurs hierarchically, from the acquisition of values and social-affective responses to context-sensitive regulation. At the first level, individuals learn emotions and social norms. Subsequently, at the second level, responses are generated based on this acquired information. Levels three and four involve processing information while considering the perspective of others, ranging from simple to complex situations. Alternatively, McDonald (2013) categorizes SC into “cold” SC, which encompasses ToM, and “hot” SC, which includes all other components. The distinction between cold and hot SC is based on the premise that rational processes shape the development of ToM. In contrast, emotional processes play a significant role in emotional processing, social knowledge, and empathy (Wilson et al., 2017).

The adequate development of SC plays a pivotal role in facilitating an individual’s social inclusion (Frith, 2008). Conversely, deficits in SC typically result in reduced social engagement and challenges in effectively integrating into society (Binder et al., 2019). SC deficits may cause an individual to experience social isolation (Spikman et al., 2012).

Acquired brain injury (ABI)—including traumatic brain injury (TBI)—is a common source of SC deficits (Maggio et al., 2022). Therefore, in addition to the traditional rehabilitation areas, such as physical or cognitive recovery, clinicians and researchers emphasize the importance of addressing deficits in social and interpersonal functioning (Turner-Stokes and Wade, 2004). Furthermore, a recent systematic review examining ToM functionality in patients with Mild Cognitive Impairment (MCI) concluded that this component of SC is often affected in all types of MCI patients, regardless of etiology and diagnostic criteria (Morellini et al., 2022). This illustrates the relationship between cognitive impairment and deficits in SC.

Additionally, research has found an association between executive functions, the affective component of ToM, and reduced awareness of dyskinesia in people with Parkinson’s Disease (PD), suggesting a complex interaction between specific neuropsychological and motor factors (Palermo et al., 2017). This connection between awareness deficits in PD and ToM further underscores the importance of understanding and addressing SC in a variety of neurological conditions, including ABI. Such insights not only expand our understanding of social cognitive processes but also emphasize the need for comprehensive interventions that address these cognitive domains in different neurological disorders.

However, it is worth noting that when clinicians evaluate individuals with ABI, SC is frequently omitted from their assessment protocol and is therefore not explicitly included in rehabilitation goals (Togher et al., 2023). Concerning this issue, Kelly et al. (2017) conducted a study involving 443 clinicians from various parts of the world to determine whether individuals with TBI had impaired SC. The results revealed that 84% of clinicians acknowledged that more than half of their TBI patients experienced SC deficits. However, 78% reported rarely or never using formal assessment tools to evaluate these domains. Sirois et al. (2019) have also emphasized the need to integrate SC assessment into the psychological evaluation protocol for individuals with ABI. These authors have further advocated for implementing tailored interventions to address SC deficits (Sirois et al., 2019).

Due to the limited use of standardized assessments, the prevalence of SC impairments following ABI remains unclear. A survey revealed that neuropsychologists lack confidence in the assessment and rehabilitation of SC, while students demand more knowledge and training in this field (Quesque et al., 2022). Wallis et al. (2022) demonstrated that in 83.11% of investigations focused on assessing SC in individuals with ABI, deficits in at least one component of this skill were reported. Regarding the type of ABI, these authors indicated that 73.7% of published studies were concerned with evaluating individuals who had experienced TBI, 15.1% on individuals who had suffered a stroke, and 20.3% did not provide explicit details about the specific characteristics of ABI within the studied samples (Wallis et al., 2022). Concerning individuals with TBI, Babbage et al. (2011) revealed that between 13 and 39% of people with moderate to severe TBI may experience significant challenges with facial affect recognition. Furthermore, SC deficits following TBI persist for at least four years in adults (Theadom et al., 2019) and two years in children (Anderson et al., 2017). Concerning individuals with stroke, Adams et al. (2019) conducted a meta-analysis of 58 datasets involving 937 stroke patients and compared them to 1,630 non-clinical controls. Their analysis concluded that three of the four core domains of social cognitive function were significantly impaired in people with stroke, specifically ToM, social knowledge, and emotional processing.

Regarding intervention approaches, the rehabilitation of SC for individuals with ABI remains relatively underdeveloped (Kelly et al., 2017) compared to similar efforts aimed at other populations, such as people with schizophrenia or autism (Cassel et al., 2016; Loubat et al., 2019). Based on a review of the training programs applied in various studies (see Supplementary Table 1 to compare components, populations, and duration of the main SC rehabilitation programs), one characteristic to highlight is that most of these rehabilitation programs focus only on one or two components of SC (Cassel et al., 2016; Loubat et al., 2019; Darling et al., 2021). Furthermore, it is noteworthy that the maximum duration of intervention is 21–24 h, as demonstrated in programs such as Theory of Mind Treatment (Steerneman et al., 1996) or SC and Interaction Training (SCIT) (Penn et al., 2005).

In summary, given its high prevalence and crucial role in facilitating effective social functioning, it is essential to explore how SC can be studied to address deficits resulting from ABI. There is currently a lack of comprehensive intervention programs targeting all SC components typically impacted after such an injury. Therefore, this study aims to address this gap by determining the effectiveness of the new “SocialMind” program in enhancing all components of SC in individuals with ABI.

## 2 Method

### 2.1 Participants

A total of 38 participants were initially recruited, 68.4% of whom were male. Nineteen participants were randomly assigned to each of the two study groups (SocialMind group and control group). Throughout the study, seven participants dropped out (one from the SocialMind group and six from the control group) due to worsening cognitive impairment in two cases, technological difficulties that hindered attendance to the online sessions in two cases, and discontinuation of attendance at their rehabilitation center in the other three cases. Consequently, the final study sample comprised 31 participants, of which 67.7% were males. The mean age of the participants was 37.44 years (22.07). Regarding the type of ABI, 48.4% had experienced traumatic brain injury (TBI), 41.9% had suffered strokes, and the remaining participants had brain tumors. Regarding the time elapsed since the injury, the control group had a mean of 10.14 (6.78) years and the experimental group 12.85 (8.80) years, with a p (0.323). No significant differences were found between the groups in terms of age, years of education, and time since injury (years), as shown in Table 1.

**TABLE 1:** Sociodemographic characteristics.

	Control Group			SocialMind Group			*t*	*p*
	N	Mean	SD	N	Mean	SD		
Age	13	37.60	16.72	18	32.66	18.86	0.759	0.578
Education (years)	13	11.47	2.59	18	12.07	3.01	0.585	0.417
Time elapsed since the injury (years)	13	10.15	6.78	18	12.85	8.80	0.943	0.323

### 2.2 Procedures

A pre/post-intervention pilot study was conducted. Participants were recruited from various rehabilitation centers specializing in acquired brain injury and hospitals throughout Spain, including the “Asociación de daño cerebral adquirido de Cádiz” (ADACCA), the “Asociación de Daño Cerebral de Granada” (AGREDACE), the “Fundación AISSE: Centro Sinergia,” and Hospital Universitario Virgen de las Nieves. Additionally, participants were recruited from private facilities through direct contact or via the project’s social media channels (116 @SocialCognicion).^1^^,^^2^^,^^3^

The inclusion criteria for this study were being between 5 and 70 years old, having suffered an acquired brain injury (ABI), and having impaired SC. ToM is widely regarded as the cornerstone of SC and, along with emotional perception and empathy, constitutes one of the most extensively researched aspects (Cassel et al., 2016; McDonald et al., 2017). Therefore, if an individual displayed impairments in this component, it likely affected their entire SC. For this reason, having impaired SC was defined as scoring fewer than 24 correct items on the MASC (Lahera et al., 2014) for adults and adolescents or fewer than 13 points on the mental inference subscale of the Everyday Life Stories (Lera-Miguel et al., 2016) for children. The exclusion criteria were having severe language or attention problems that limited the patient’s capacity to take part in the intervention.

**FIGURE 1:**
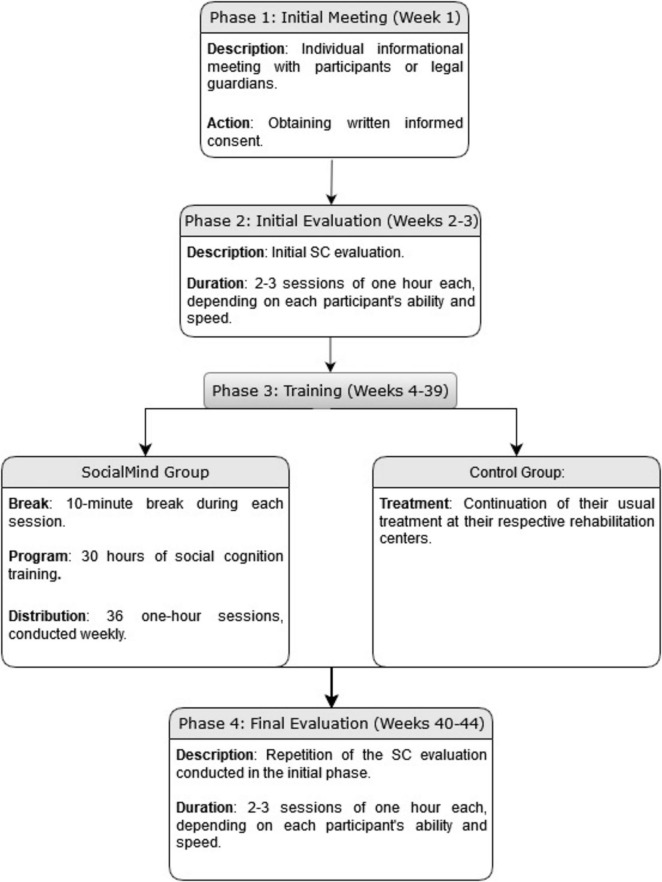
SocialMind treatment flowchart.

The study lasted 44 weeks for each participant, divided into four distinct phases: (1) initial meeting, (2) initial evaluation, (3) training, and (4) final evaluation. All activities were administered individually. First, an individual information meeting was held with the potential participants or legal guardians to provide a comprehensive overview of the study. Written informed consent was obtained from all participants or their legal guardians to participate in the study. Second, the initial SC evaluation was carried out and repeated at the end of the training. The duration of these evaluations averaged between two to three sessions, each lasting for 1 h, depending on the ability and speed of each participant. In the third phase, the SocialMind group engaged in a training program lasting for 30 h, distributed across 36 1-h sessions, conducted weekly, with a 10-min break during each session. Participants in the control group continued their treatment as usual at their respective rehabilitation centers. Regarding the intervention procedure, participants utilized a computer for the tasks and responded orally to the questions. Feedback was provided as part of the sessions. The study was conducted following the Declaration of Helsinki and approved by the Human Research Ethics Committee of the University of Granada (N°certificate 706/CEIH/2018). Figure 1 shows a SocialMind treatment flowchart.

### 2.3 Material

#### 2.3.1 Assessment of emotional processing

Emotional processing was evaluated using Baron Cohen’s face test (Baron-Cohen et al., 1997), specifically the Spanish version developed by Huerta-Ramos et al. (2021). This test evaluates the ability to recognize facial emotional expressions by presenting twenty images of an actress depicting both basic emotions—happiness, sadness, fear, surprise, disgust, and distress—and complex mental states, including scheming, guilt, thoughtfulness, admiration, quizzical, flirtatiousness, boredom, interest, and arrogance. This test is suitable for children, adolescents, and adults (Dziobek et al., 2005; Huerta-Ramos et al., 2021). The overall score is computed as the sum of correct responses and ranges between 0 and 20.

#### 2.3.2 Assessment of social knowledge

For the assessment of social knowledge, the Faux pas test was administered. The children’s version, developed by Baron-Cohen et al. (1999), was employed for participants up to the age of 15, while the adult version developed by Stone et al. (1998) adapted for Spanish adults by Fernández-Modamio et al. (2018) was used for participants aged 16 and above. Both versions of the Faux pas test consist of ten stories that include faux pas situations and ten control stories. In the adult version, each story requires participants to answer eight questions regarding their ability to detect faux pas, understand inappropriateness, interpret the speaker’s intention, evaluate associated false beliefs, consider related emotions/empathy, and respond to control questions to ensure they understand the story. In the children’s version, participants have to answer four questions on faux pas detection, identification, comprehension, and false beliefs for each story. The participant is awarded one point for each story if they answer all the questions correctly. A score of 0 is awarded if any of the responses to the question were incorrect. Consequently, the total score can vary between 0 and 20, considering there are a total of 20 stories (10 control and 10 faux pas), with one point allocated to each narrative

#### 2.3.3 Assessment of ToM

ToM was evaluated using the Movie for the Assessment of Social Cognition (MASC) (Dziobek et al., 2006) in its Spanish version developed by Lahera et al. (2014) in participants aged 16 and above. The MASC involves a film featuring four characters spending a Saturday night together. Participants are asked to make inferences about the mental states of the characters depicted in the video. The test addresses different modalities of mental states, including thoughts, emotions, and intentions, with neutral, positive, and negative valence. Participants are presented with 45 questions, each with four response options, of which only one is correct. The questions appear immediately after each scene in the movie, and the video is programmed to pause until the participant selects their answer. Participants are required to choose one response from four options: a correct attribution of ToM to the characters, an overmentalizing error (a mental state that is attributed when there is no reason for it), an undermentalizing error (failing to attribute a present mental state when it is warranted), or a total absence of mentalizing error (attributing of physical causality rather than a mental state). The summed score across all of the responses yields four complementary partial scores: the number of correct responses, the number of errors due to overmentalizing, the number of errors due to undermentalizing, and the number of errors due to the absence of mentalizing. The present study used the overall number of correct responses, with the score ranging from 0 to 45.

Stories of Everyday Life (Kaland et al., 2002), in its Spanish version developed by Lera-Miguel et al. (2016), was administered to measure ToM in children aged between 7 and 15. This instrument includes 13 types of stories: lies, white lies, figure of speech, misunderstanding, double entendre, irony, persuasion, contrary emotions, forgetting, jealousy, intentions, empathy, and social blunders. Each story has between 10 and 15 control questions to ensure participants understand the story. Additionally, each story has three essential questions to assess the participant’s ability to infer the characters’ mental and physical states from the story’s context. Responses are scored as correct (2 points), partially correct (1 point), or incorrect (0 points). The present study used the overall score obtained on the mental inference subscale, ranging from 0 to 26 points.

#### 2.3.4 Assessment of empathy

Each participant was given an age-appropriate empathy test. For adults, we employed the Empathy Quotient (EQ) test developed by Baron-Cohen and Wheelwright (2004), consisting of 60 self-reported items. Of these, 40 items were related to empathy (e.g., I can easily tell if someone else wants to enter a conversation), and the remaining 20 items served as controls. Participants in the adolescent and child categories were evaluated using the versions of the test adapted to their age groups. We used the adolescent version of the test developed by Auyeung et al. (2012), comprising 40 items, and the children’s version by Auyeung et al. (2009), which included 55 items for parents to assess their child’s empathy levels. Parents were asked to agree or disagree with each statement concerning their child (e.g., My child often doesn’t understand why some things upset other people so much).

In both versions, there were four response options (strongly agree, slightly agree, slightly disagree, and strongly disagree). To minimize response bias, half of the items are phrased so that agreement indicates empathy, while for the other half, disagreement indicates empathy. An absence of empathy receives a score of zero, a slight empathy response scores one point, and a strong empathy response scores two points. An individual can obtain a score of 2, 1, or 0 points for each empathy-related item. The overall score is calculated by summing the scores across all items.

#### 2.3.5 Intervention

The SocialMind rehabilitation program was specifically designed for this study with a focus on enhancing the four core SC components: emotion processing, social knowledge, ToM, and empathy. The program was implemented online but is equally suitable for in-person and remote delivery. It lasts for 30 h, with weekly sessions, each lasting 1 h and including a 10-min break.

The program comprises four modules, each targeting one of the SC components. The first module is centered on emotion processing, where participants will (1) learn to recognize different emotions, (2) distinguish between positive and negative emotions, (3) associate each emotion with its corresponding facial expression, and (4) analyze real-life situations to determine whether the observed facial expressions matched the emotion being felt. The second module emphasizes social awareness, where participants will (1) understand the social norms of various situations, (2) identify the appropriate behaviors of various characters, and (3) analyze real-life scenarios in which they (the participants) did not conform to social norms. The third module is dedicated to ToM and covers (1) the notion that different people can have varying perspectives, (2) an understanding of indirect language, and (3) the identification and comprehension of irony. Finally, the fourth module focuses on empathy, helping participants recognize the importance of considering others’ feelings before speaking or acting. To achieve this, participants are asked to (1) assess the behavior of various characters and determine whether the action is empathetic, (2) reflect on personal situations where they did not act empathetically, and (3) propose alternative actions in place of non-empathetic behaviors.

**FIGURE 2:**
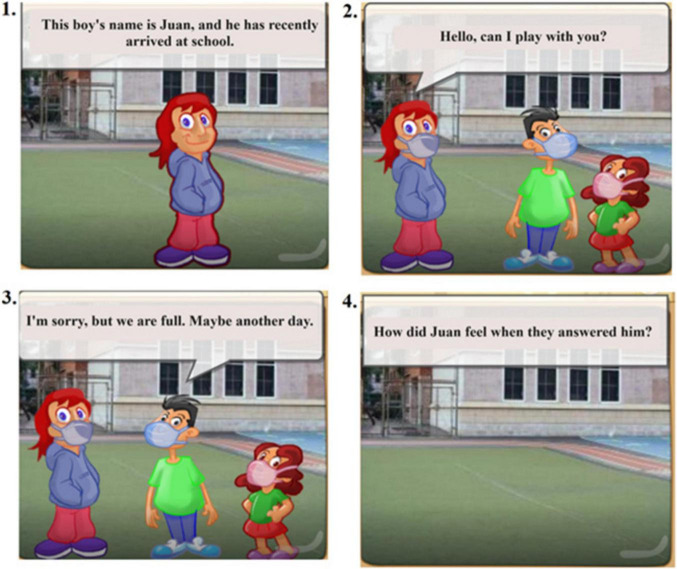
Example translation to English of a ToM exercise created with the Mobbyt application.

**FIGURE 3:**
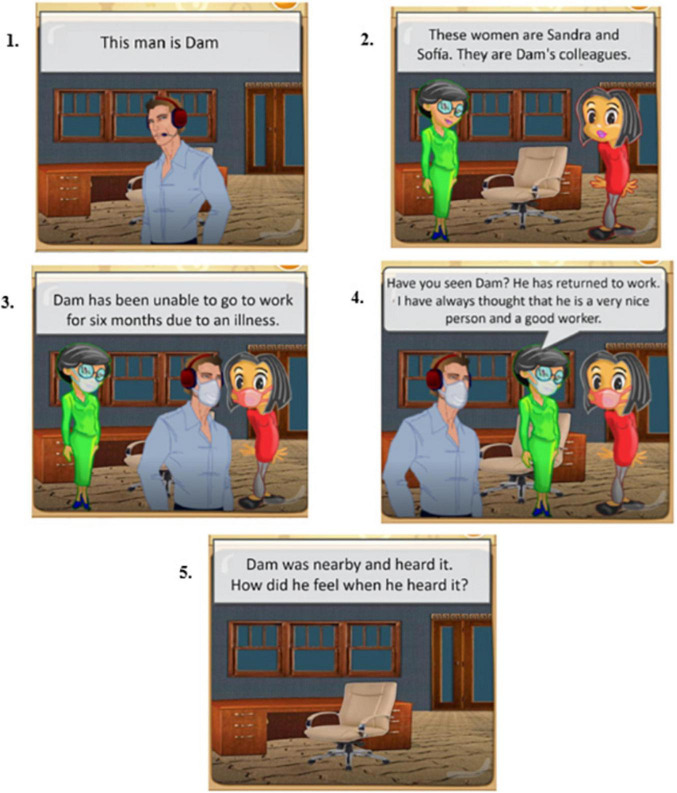
Illustrative Translation into English of a Theory of Mind (ToM) Exercise Generated with the Mobbyt Application (for Children aged 15 and above).

The training program utilizes various resources, including original stories, images obtained from free sources such as Pixabay or Pexels, videos/movies, and free apps for creating online games such as mobbyt.^4^ All tasks are adapted to children, adolescents, and adults. Figures 2, 3 shows an example exercise corresponding to the block dedicated to ToM training for children under 15 years and above. The complete program is in the process of being published as a handbook. For further information on the exercises, please contact the authors.

### 2.4 Statistical analysis

The raw scores for each instrument were transformed into standard scores (Z). These standard scores for the various forms of each test (child, adolescent, and adult versions) were then pooled to form a single variable for each SC component. For instance, the ToM variable was created by combining the Z scores from the MASC hit score (Dziobek et al., 2006) for adolescent and adult participants and the Everyday Life Stories (Kaland et al., 2002) score for children.

Non-parametric tests were employed due to the limited sample size. Specifically, the Mann-Whitney U-test was conducted to compare the two groups. This comparison was based on the individual changes for each participant, which were calculated using the following formula: score at post-intervention minus score at the pre-intervention. The effect size for the Mann-Whitney U-test was calculated using the Partial Eta Squared measure, following Lenhard and Lenhard (2016). The data were analyzed using SPSS Statistics version 28 (IBM Corp, 2021).

## 3 Results

Table 2 shows the mean scores (and standard deviations) for each group at the two assessment points. Between-group analyses (Table 3) revealed a statistically significant improvement in the experimental group compared to the control group in emotional processing, social knowledge, and empathy.

**TABLE 2:** Scores pre and post intervention.

	SocialMind Group		Control Group	
	Pre Mean (SD)	Post Mean (SD)	Pre Mean (SD)	Post Mean (SD)
Emotional processing	14.06 (2.16)	15.44 (1.98)	16.00 (3.48)	15.15 (3.21)
Social knowledge	10.89 (5.53)	16.28 (2.82)	14.23 (3.35)	14.31 (3.45)
ToM	9.50 (6.45)	14.72 (7.60)	16.62 (5.59)	18.23 (8.34)
Empathy	27.22 (10.95)	34.00 (16.83)	40.54 (10.17)	36.46 (7.76)

Note. Emotion recognition: Baron Cohen’s face test; Social knowledge: Faux pas; ToM: Movie for the Assessment of Social Cognition (MASC) and Stories of Everyday Life; Empathy: Empathy Quotient.

**TABLE 3:** Results from individual change between groups.

	SocialMind Group	Control Group	Mann-Whitney test			Effect size
	Mean Range	Mean Range	*U*	*Z*	*p*	η^2^
Emotional processing	19.28	11.46	58.00	−2.38	0.017*	0.315
Social knowledge	20.81	9.35	30.50	−3.48	< 0.001*	0.673
ToM	18.64	12.35	69.50	−1.91	0.057	0.203
Empathy	20.58	9.65	34.50	−3.30	0.001*	0.605

Emotion recognition: Baron Cohen’s face test; Social knowledge: Faux pas; ToM: Movie for the Assessment of Social Cognition (MASC) and Stories of Everyday Life; Empathy: Empathy Quotient; η^2^: Partial Eta squared.

*Indicates significant values.

## 4 Discussion

This study aimed to assess the effectiveness of the SocialMind program in enhancing SC in individuals with ABI. The results revealed significant improvements in emotion recognition, social knowledge, and empathy in the SocialMind group compared with controls. Additionally, marginally significant improvements in ToM were observed. The effect sizes for all SC components were large according to the guidelines established by Cohen et al. (2003).

In the context of rehabilitation, the practical implications of these effect sizes are particularly noteworthy. Effect size provides valuable information beyond statistical significance, especially in pilot studies with small sample sizes. Large effect sizes, as observed in our study, suggest that the SocialMind program impact on improving SC components, which might be crucial for the social and functional reintegration of individuals with ABI (Adolphs, 2010; McDonald and Genova, 2021). From a clinical perspective, these improvements might imply better social interactions, conflict resolution, and establishing meaningful relationships (Spikman et al., 2012) as a way to fight against the risk of isolation and depression (Kuiper and Higgins, 1985). However, it is important to recognize that while large effect indicate substantial improvements, they do not inherently convey clinical significance. The real-world impact of these improvements must be evaluated in terms of how they enhance daily functioning and social interactions. Practically speaking, the effectiveness of the SocialMind program is particularly relevant given the scarcity of SC rehabilitation programs specifically designed for individuals with ABI but clinicians and future studies must show the efficacy in the patients’ real life.

In contrast, certain training programs targeting various components of SC in other populations have had limited success. For example, Social Cognition and Interaction Training, a 24-session program designed for individuals with schizophrenia, includes exercises focused on emotion recognition and ToM. However, reports suggest that the program was only effective in enhancing ToM, but not emotional recognition (Penn et al., 2005). Similarly, the Social Cognitive Skills Training program, consisting of two six-session modules, yielded significant improvements in emotional processing, but not ToM, in individuals with schizophrenia (Horan et al., 2009). Finally, Virtual Reality Social Cognition Training, designed for individuals with autism, enhanced emotional processing but could not improve ToM (Kandalaft et al., 2013).

Among the few rehabilitation programs aimed at enhancing SC for individuals with ABI, Radice-Neumann et al. (2009) conducted a study to improve emotional recognition and ToM. While participants showed enhanced emotion recognition, they did not show significant improvements in the ability to infer the mental states of others. The Treatment for Impairments in Social Cognition and Emotion Regulation (T-ScEmo) improved both emotion recognition and ToM but not social knowledge (Westerhof-Evers et al., 2017). Finally, the Computerized Social Cognitive Training program yielded significant improvements in ToM, but not emotion recognition (Rodríguez-Rajo et al., 2022).

Our findings highlight the innovative nature of the SocialMind training program. Not only does this program cater to individuals with ABI, a population for whom relatively few SC rehabilitation programs are available (Vallat-Azouvi et al., 2019), but it is also the only program that has demonstrated effectiveness in improving more than two SC components. However, it is important to note that while the effect size for ToM was large, the changes only approached statistical significance. This finding might suggest that the methodology employed to improve ToM should differ from that used for other SC components. This distinction follows from the model that categorizes SC components into “cold” cognition, involving more cognitive and detached processes, and “hot” cognition, which encompasses more emotionally charged and context-dependent processes (McDonald, 2013). For this reason, at the end of each module, the SocialMind program included exercises focused on emotional processes dedicated to the “hot” SC components, where participants were asked to provide real-life examples of their experiences. Moreover, it is crucial to consider that brain networks involved in social cognition, such as the social perception network, mirror network, emotional networks, and mentalizing network, interact synergistically to support social processing (Abalo-Rodríguez et al., 2023). The social perception network is dedicated to processing and interpreting signals from the social context, while the mirror network observes and imitates others’ actions. Emotional networks assess stimulus relevance, detect threats, and foster empathy, whereas the mentalizing network underpins Theory of Mind processes (Abalo-Rodríguez et al., 2023). These networks, with their specific brain regions, are essential for understanding how programs like SocialMind can influence rehabilitation following acquired brain injuries. Moreover, they explain why outcomes in Theory of Mind may differ from other components, as they engage different brain networks.

Furthermore, in terms of training duration, it is worth noting that the T-ScEmo program developed by Westerhof-Evers et al. (2017) allocates half of its total intervention time to the ToM block, suggesting that rehabilitation of the ToM component requires more time than the rest (this capacity is underpinned by unique processes that are not targeted in the rehabilitation of other skills). Therefore, future research should consider increasing the time dedicated to training this specific component.

From a clinical perspective, the persistence of difficulties in social cognitive areas can lead to social isolation, depression, and a decrease in the quality of life for individuals and their families (Kuiper and Higgins, 1985). Therefore, the improvements observed after the SocialMind program have been associated in other studies with significant enhancements in the social and functional reintegration of individuals with ABI (Adolphs, 2010; McDonald and Genova, 2021). Positive changes in SC are crucial for better social interaction, conflict resolution, and the establishment of meaningful relationships (Spikman et al., 2012).

From a practical standpoint, therapists often find themselves without validated tools to address the rehabilitation of complex aspects of SC. The SocialMind program is particularly valuable and specific because it has demonstrated its efficacy for individuals with ABI. Approaching the rehabilitation of SC can significantly improve patients’ ability to reintegrate socially.

There are many examples of individuals who could benefit from the SocialMind intervention. For instance, people who have difficulties recognizing emotions in others and understanding their thoughts and feelings often experience negative social interactions. After completing the Emotion Recognition, Social Knowledge, and Empathy modules of the program, these individuals will experience considerable improvement in understanding their own emotions and accurately predicting others’ intentions and behaviors. These changes can make them feel more confident and competent in personal interactions and enable them to integrate more actively into their communities.

## 5 Conclusion

The SocialMind program offers a valuable and innovative approach to enhancing SC in individuals with ABI. The results of this study demonstrate the program’s effectiveness in improving three of the four key components of this ability.

## 6 Limitations and strengths

This study has several limitations that need to be considered. Firstly, the sample size was relatively small, which limits the generalizability of the results. For future research, it is recommended to increase the number of participants to enhance the external validity of the study. Secondly, the control group did not receive a placebo treatment, which may have influenced the results. To ensure comparability between groups, it is crucial to include a placebo treatment in future studies.

Third, detailed information related to specific details about the type of ABI as the location and size of the brain lesions. This information is crucial for a comprehensive understanding of the study context and its implications for SC. Future research should strive to include these parameters to provide more complete and valuable insights into the impact of acquired brain injuries

Additionally, in the present study, pre-existing cognitive levels and a baseline assessment of cognitive functioning were not considered. These factors are crucial in determining whether there are underlying mechanisms that might have affected SC. Specifically, aspects such as visuoperceptual abilities, attention capacity, working memory, language comprehension, and mood tone should be evaluated. In future research, these aspects will be considered and included.

Lastly, regarding the limitations in the assessment instruments for SC and its components, there are few tools specifically designed for this purpose (Tousignant et al., 2018) and they must be culturally adapted (Wellman et al., 2006). Given this need, the present study had few options to choose from. However, to overcome this limitation, we selected instruments that had already been specifically adapted in other studies to the Spanish population.

Despite these limitations, the study presents positive aspects, such as the introduction of a comprehensive rehabilitation program for SC. The flexibility and accessibility of the program, which can be implemented both in-person and online, are noteworthy and suggest its potential usefulness for individuals with acquired brain injuries.

## Data availability statement

The raw data supporting the conclusions of this article will be made available by the authors, without undue reservation.

## Ethics statement

The studies involving humans were approved by the Human Research Ethics Committee of the University of Granada (certificate no. 706/CEIH/2018). The studies were conducted in accordance with the local legislation and institutional requirements. Written informed consent for participation in this study was provided by the participants’ legal guardians/next of kin.

## Author contributions

SR-G: Conceptualization, Data curation, Formal analysis, Investigation, Methodology, Writing−original draft. OG-B: Writing−review and editing. ANC: Writing−review and editing. ALC: Formal analysis, Investigation, Methodology, Supervision, Writing−original draft.

## Footnotes

1
https://n9.cl/1b6de

2
https://acortar.link/GNBDB9

3
https://acortar.link/O4TMb2

4
https://mobbyt.com
