# Supplementary material for: Pilot study on the effectiveness of the socialmind program for the rehabilitation of social cognition following acquired brain injury
Source: Front Psychol. 2024 Jul 17;15:1338335. doi: 10.3389/fpsyg.2024.1338335 (PMC11288943; doi:10.3389/fpsyg.2024.1338335)
Supplement: Supplementary file 1 [file Table_1.DOCX]

Supplementary Material

# Improving interactions with others after traumatic brain injury: a single-case experimental study

Sandra Rivas-García^1,2*^, Olga García-Bermúdez^3^, Andrés Catena^2,4^ and Alfonso Caracuel^2,5^

*** Correspondence:**

Sandra Rivas García. Faculty of Education Sciences, 11519 Puerto Real, Cádiz.

E-mail: srivasresearcher@gmail.com

# Supplementary Figures and Tables

| **Table 1** | | | |
| --- | --- | --- | --- |
| *Social cognition intervention programs (in chronological order of publication)* | | | |
| **Training** | **SC Components** | **Population** | **Duration of training** |
| Theory of Mind Treatment (Steerneman et al., 1996) | Emotional Processing | Autism Spectrum Disorder | 21 session/1 hour |
| Training of Affect Recognition (TAR) (Frommann et al., 2003) | Emotional Processing | Schizophrenia Spectrum Disorder | 12 session/45 min. |
| Emotion Management Training (EMT) (Hodel et al., 2004) | Emotional Processing | Schizophrenia Spectrum Disorder | 24 session/45 min. |
| Metacognitive and social cognition training (MSCT) (Hogarty et al., 2004) | Emotional Processing  Theory of Mind | Schizophrenia Spectrum Disorder | 18 session/1 hour |
| Social cognition and Interaction Training (SCIT) (Penn et al., 2005) | Emotional Processing  Theory of Mind | Schizophrenia Spectrum Disorder | 24 session/1 hour |
| Mind-Reading: Interactive Guide to Emotions (Golan & Baron-Cohen, 2006) | Emotional Processing | Autism Spectrum Disorder | 20 session/1 hour |
| Metacognitive Training (MCT) (Moritz & Woodward, 2007) | Theory of Mind | Schizophrenia Spectrum Disorder | 8 session/1 hour |
| Micro Expression Training Tool (METT) (Russell et al., 2008) | Emotional Processing | Schizophrenia Spectrum Disorder | 12 session/1 hour |
| Social Cognitive Social Knowledgeill Training (SCST) (Horan et al., 2009) | Emotional Processing  Social Knowledge  Theory of Mind | Schizophrenia Spectrum Disorder | 12 session/45 min. |
| Training Emotional Processing (Radice-Neumann et al., 2009) | Emotional Processing  Theory of Mind | Traumatic Brain Injury | 9 session/1 hour |
| Emotion and Theory of Mind Imitation Training (Mazza et al., 2010) | Emotional Processing  Theory of Mind | Schizophrenia Spectrum Disorder | 8 session/50 min. |
| Reading a Smile: An Emotion PercEmotional Processing Treatment Program (Bornhofen & McDonald, 2010) | Emotional Processing | Traumatic Brain Injury | 16 session/ 90 min. |
| Mind Reading: An Interactive Guide to Emotions (MRIGE) (Lindenmayer et al., 2013) | Emotional Processing | Schizophrenia Spectrum Disorder | 12 session/1 hour |
| Virtual Reality Social Cognition Training (VR-SCT) (Kandalaft et al., 2013) | Emotional Processing  Social Knowledge  Theory of Mind | Autism Spectrum Disorder | 10 session/1 hour |
| Intervention for deficits in recognizing emotional prosody (McDonald et al., 2013) | Emotional Processing | Traumatic Brain Injury | 3 session/ 2 hours |
| SocialVille (Nahum et al., 2014) | Emotional Processing  Social Knowledge  Theory of Mind | Schizophrenia Spectrum Disorder | 24 hours |
| Emotion recognition training (Neumann et al., 2015) | Emotional Processing | Traumatic Brain Injury | 9 session/ 1 hour |
| E-motional Training (Vázquez-Campo et al., 2016) | Complete Social Cognition | Schizophrenia Spectrum Disorder | 12 hours |
| Treatment for Impairments in Social Cognition and Emotions Regulations (T-ScEmo) (Westerhof-Evers et al., 2017) | Emotional Processing  Social Knowledge  Theory of Mind | Traumatic Brain Injury | 20 session/1 hour |
| Computerized social cognitive training (Rodriguez-Rajo et al., 2022) | Complete Social Cognition | Traumatic Brain Injury | 21 session/1 hour |

# Reference

Bornhofen, C., & McDonald, S. (2010). *Reading a Smile (and Other Great Expressions): An Emotion Perception Treatment Program*. Australian Society for the Study of Brain Impairment. ASSBI Resources.

Frommann, N., Streit, M., & Wölwer, W. (2003). Remediation of facial affect recognition impairments in patients with schizophrenia: A new training program. *Psychiatry Research*, *117*(3), 281–284. https://doi.org/10.1016/S0165-1781(03)00039-8

Golan, O., & Baron-Cohen, S. (2006). Systemizing empathy: Teaching adults with Asperger syndrome or high-functioning autism to recognize complex emotions using interactive multimedia. *Development and Psychopathology*, *18*(2), 591–617. https://doi.org/10.1017/S0954579406060305

Hodel, B., Kern, R. S., & Brenner, H. D. (2004). Emotion Management Training (EMT) in persons with treatment-resistant schizophrenia: First results. *Schizophrenia Research*, *68*(1), 107–108. https://doi.org/10.1016/S0920-9964(03)00119-1

Hogarty, G. E., Flesher, S., Ulrich, R., Carter, M., Greenwald, D., Pogue-Geile, M., Kechavan, M., Cooley, S., DiBarry, A. L., Garrett, A., Parepally, H., & Zoretich, R. (2004). Cognitive Enhancement Therapy for Schizophrenia: Effects of a 2-Year Randomized Trial on Cognition and Behavior. *Archives of General Psychiatry*, *61*(9), 866–876. https://doi.org/10.1001/archpsyc.61.9.866

Horan, W. P., Kern, R. S., Shokat-Fadai, K., Sergi, M. J., Wynn, J. K., & Green, M. F. (2009). Social cognitive skills training in schizophrenia: An initial efficacy study of stabilized outpatients. *Schizophrenia Research*, *107*(1), 47–54. https://doi.org/10.1016/j.schres.2008.09.006

Kandalaft, M. R., Didehbani, N., Krawczyk, D. C., Allen, T. T., & Chapman, S. B. (2013). Virtual Reality Social Cognition Training for Young Adults with High-Functioning Autism. *Journal of Autism and Developmental Disorders*, *43*(1), 34–44. https://doi.org/10.1007/s10803-012-1544-6

Lindenmayer, J.-P., McGurk, S. R., Khan, A., Kaushik, S., Thanju, A., Hoffman, L., Valdez, G., Wance, D., & Herrmann, E. (2013). Improving Social Cognition in Schizophrenia: A Pilot Intervention Combining Computerized Social Cognition Training With Cognitive Remediation. *Schizophrenia Bulletin*, *39*(3), 507–517. https://doi.org/10.1093/schbul/sbs120

Mazza, M., Lucci, G., Pacitti, F., Pino, M. C., Mariano, M., Casacchia, M., & Roncone, R. (2010). Could schizophrenic subjects improve their social cognition abilities only with observation and imitation of social situations? *Neuropsychological Rehabilitation*, *20*(5), 675–703. https://doi.org/10.1080/09602011.2010.486284

McDonald, S., Togher, L., Tate, R., Randall, R., English, T., & Gowland, A. (2013). A randomised controlled trial evaluating a brief intervention for deficits in recognising emotional prosody following severe ABI. *Neuropsychological Rehabilitation*, *23*(2), 267–286. https://doi.org/10.1080/09602011.2012.751340

Moritz, S., & Woodward, T. S. (2007). Metacognitive training in schizophrenia: From basic research to knowledge translation and intervention. *Current Opinion in Psychiatry*, *20*(6), 619–625. https://doi.org/10.1097/YCO.0b013e3282f0b8ed

Nahum, M., Fisher, M., Loewy, R., Poelke, G., Ventura, J., Nuechterlein, K. H., Hooker, C. I., Green, M. F., Merzenich, M. M., & Vinogradov, S. (2014). A novel, online social cognitive training program for young adults with schizophrenia: A pilot study. *Schizophrenia Research: Cognition*, *1*(1), e11–e19. https://doi.org/10.1016/j.scog.2014.01.003

Neumann, D., Babbage, D. R., Zupan, B., & Willer, B. (2015). A randomized controlled trial of emotion recognition training after traumatic brain injury. *The Journal of Head Trauma Rehabilitation*, *30*(3), E12-23. https://doi.org/10.1097/HTR.0000000000000054

Penn, D., Roberts, D. L., Munt, E. D., Silverstein, E., Jones, N., & Sheitman, B. (2005). A pilot study of social cognition and interaction training (SCIT) for schizophrenia. *Schizophrenia Research*, *80*(2), 357–359. https://doi.org/10.1016/j.schres.2005.07.011

Radice-Neumann, D., Zupan, B., Tomita, M., & Willer, B. (2009). Training emotional processing in persons with brain injury. *The Journal of Head Trauma Rehabilitation*, *24*(5), 313–323. https://doi.org/10.1097/HTR.0b013e3181b09160

Rodriguez-Rajo, P., Garcia-Rudolph, A., Sanchez-Carrion, R., Aparicio-Lopez, C., Ensenat-Cantallops, A., & Garcia-Molina, A. (2022). Computerized social cognitive training in the subacute phase after traumatic brain injury: A quasi-randomized controlled trial. *APPLIED NEUROPSYCHOLOGY-ADULT*. https://doi.org/10.1080/23279095.2022.2042693

Russell, T. A., Green, M. J., Simpson, I., & Coltheart, M. (2008). Remediation of facial emotion perception in schizophrenia: Concomitant changes in visual attention. *Schizophrenia Research*, *103*(1–3), 248–256. https://doi.org/10.1016/j.schres.2008.04.033

Steerneman, P., Jackson, S., Pelzer, H., & Muris, P. (1996). Children with Social Handicaps: An Intervention Programme Using a Theory of Mind Approach. *Clinical Child Psychology and Psychiatry*, *1*(2), 251–263. https://doi.org/10.1177/1359104596012006

Vázquez-Campo, M., Maroño, Y., Lahera, G., Mateos, R., & García-Caballero, A. (2016). e-Motional Training®: Pilot study on a novel online training program on social cognition for patients with schizophrenia. *Schizophrenia Research: Cognition*, *4*, 10–17. https://doi.org/10.1016/j.scog.2015.11.007

Westerhof-Evers, H. J., Visser-Keizer, A. C., Fasotti, L., Schönherr, M. C., Vink, M., van der Naalt, J., & Spikman, J. M. (2017). Effectiveness of a Treatment for Impairments in Social Cognition and Emotion Regulation (T-ScEmo) After Traumatic Brain Injury: A Randomized Controlled Trial. *Journal of Head Trauma Rehabilitation*, *32*(5), 296–307. https://doi.org/10.1097/HTR.0000000000000332
